# Achieving HIV epidemic control through accelerating efforts to expand access to pre-exposure prophylaxis for people who inject drugs

**DOI:** 10.3389/frph.2024.1438005

**Published:** 2024-10-23

**Authors:** Lirica Nishimoto, Adaobi Lisa Olisa, Philip Imohi, Judy Chang, Chris Obermeyer, Dama Kabwali, Christopher Akolo

**Affiliations:** ^1^ FHI 360, Global Programs and Science Department, Washington, DC, United States; ^2^ FHI 360, Global Programs and Science Department, Abuja, Nigeria; ^3^ International Network of People who Use Drugs, London, England; ^4^ The Global Fund to Fight AIDS, Tuberculosis and Malaria, Geneva, Switzerland; ^5^ Tanzania Network for People who Use Drugs, Dar es Salaam, Tanzania

**Keywords:** PWID, HIV, PrEP, prevention, integration, community-led, access, new and emerging products

## Abstract

The world is not on track to reach the majority of the UNAIDS 2025 targets, and people who inject drugs (PWID) continue to be left behind, hindered by counterproductive law enforcement practices, punitive laws, economic distress, and social stigma and discrimination. Poor access to HIV pre-exposure prophylaxis (PrEP) among PWID is nested within the limited access to broader harm reduction services, including needle and syringe programs, opioid overdose management, opioid agonist therapy (also known as medication-assisted treatment), and condoms. Among PWID, women who inject drugs are disproportionately affected and face additional gender-based barriers. Intersections between PWID and other key and priority population groups also exist. Although the prioritization of PWID for new PrEP products like the dapivirine vaginal ring and injectable cabotegravir has lagged in research, studies have shown that PWID find injectable and long-acting options acceptable and preferrable, including among women who inject drugs. While new PrEP products introduce new opportunities, equity in access must be assured for optimized impact toward achieving epidemic control. Programming for services must engage and empower PWID community leadership to address the structural barriers to services, implement community-led, differentiated, and integrated service modalities, and offer the choice of all harm reduction options to close the equity gaps in health outcomes. While waiting for necessary evidence and approvals, programs should work together with the PWID community to prioritize, expand, and facilitate efforts and investments toward increased access to and integration of PrEP and all recommended harm reduction services for PWID.

## Introduction

Although great strides have been made, the world is not on track to reach many of the Joint United Nations Programme on HIV/AIDS (UNAIDS) 2025 targets, including reducing new HIV acquisitions to under 370,000 and ensuring oral pre-exposure prophylaxis (PrEP) availability for 10 million people at higher likelihood of acquiring HIV (1). Globally in 2022, there were 1.3 million new HIV diagnoses, and 2.5 million people received PrEP at least once (1). Gaps in access to prevention services, including oral PrEP, are specifically heightened among key and priority populations, including people who inject drugs (PWID) (1). As new PrEP products are rolled out, it is imperative that enduring gaps in access to prevention services among PWID be addressed and inequalities in access be diminished.

## HIV and other epidemics among PWID

Globally, there were around 13.2 million PWID in 2021 (2). Accounting for 10% of global new HIV acquisitions (2), HIV prevalence among members of this population is estimated to be seven times higher compared to the general population (1), and their likelihood of acquiring HIV is 35 times higher (2). While PWID have one of the largest gaps among key population groups in country-reported size estimates, the antiretroviral therapy (ART) coverage is estimated to be 69%, and viral load suppression rates are estimated to be low, resulting in high HIV transmission rates (1). Therefore, significant efforts at scale-up of HIV prevention interventions are necessary.

In addition to HIV, PWID also have an increased likelihood of exposure to hepatitis B virus (HBV) and hepatitis C virus (HCV) (2). Global estimates indicate that among PWID who are living with HIV, 8.4% have current HBV infection, and 38.8% have current HCV infection (3). In addition, 23% of new HCV infections and one in three HCV-related deaths are attributable to injecting drug use (4).

## Low access to recommended prevention services for PWID

While PrEP has been recommended for all individuals at increased likelihood of acquiring HIV, access remains poor across most countries and populations, including among PWID. Poor access to PrEP among PWID is nested within limited access to broader harm-reduction (HR) services that have been proven effective in improving health outcomes among this population, including needle and syringe programs (NSPs) (5), opioid overdose management (OOM), opioid agonist therapy (OAT), also known as medication-assisted treatment (MAT), and condoms. OAT is operational in 90 countries, mostly on a small scale with only a handful of countries reaching at least 40 people with OAT services per 100 PWID, and a global estimate of 18 people reached per 100 PWID (6). Only 12 have achieved 90% coverage of safe injecting practices (1). Condom distribution programs for PWID exist in 42 countries, with six countries specifically stating that condom distribution does not target PWID (7).

Oral PrEP for HIV was not originally recommended for PWID, partially due to some reservations voiced among the PWID community, including the need to focus first on sufficiently expanding access to HIV treatment before redirecting investments to oral PrEP and the fear of oral PrEP replacing NSP, OAT, OOM, and other proven interventions that improve health outcomes beyond HIV (8). Prioritization, expansion, and facilitation of access to all recommended services for PWID are necessary to foster trust and acceptability of new promising products and interventions within the community and, therefore, access to comprehensive care.

While evidence shows injecting drug use to be occurring in 190 countries (6), oral PrEP services for PWID have been identified in only 27, all of which were small in scale and limited in geographic coverage and service locations (9). Access to services among PWID is also impeded by structural barriers including criminalization, stigma, and discrimination.

## Improving access to PrEP as part of a comprehensive package of care

Oral PrEP is recommended for and preferred within the PWID community as one option among several options for HR (7, 10). Oral PrEP cannot replace other HR options because it only prevents HIV acquisition and does not address other health concerns, including HCV and HBV. A randomized controlled trial conducted in Bangkok, Thailand with people who reported injecting drug use in the past year found that PrEP use among PWID effectively reduced their likelihood of acquiring HIV by 48.9% (11), but the generalizability of the findings was limited. The implementation of daily oral PrEP with optimal adherence may not be practical for all PWID in all contexts due to the multiple social and legal barriers to access and varying individual circumstances (12), further highlighting the need to offer PrEP along with all options of HR through tailored services. Moreover, the lack of information about and coordination among services has been cited as a barrier to care among PWID (13, 14). The scale-up of HR services in general is necessary for the accessible and acceptable scale-up of PrEP.

Where oral PrEP is available for PWID currently, the offering often remains within vertical HIV programming, and services are often provided without direct links to other HR services (6). Advocacy for and implementation of tailored, person-centered care for PWID and expanded coverage of integrated PrEP, OAT, OOM, NSP, condoms, and other HR services is needed to the extent possible rather than an expansion of siloed services. In addition, this advocacy should include services beyond HIV, such as other associated communicable and noncommunicable diseases and broader health and social services. Furthermore, expanding access to HIV treatment to ensure HIV viral suppression and the reduced likelihood of transmission cannot be neglected.

## Programming to address structural barriers to create an enabling environment

Several barriers have been found to hinder access to health care among people who use and inject drugs. One common barrier is stigma and discrimination from health care providers (1, 8, 12, 13, 15). A survey found that more than 10% of PWID avoided accessing health care services due to stigma and discrimination in eight of the 14 countries reporting to the UNAIDS (1).

Harassment from law enforcement, counterproductive law enforcement practices, and punitive laws have also been reported as barriers to PWID's access to care (1, 8, 12, 13, 14). While strong evidence links the criminalization of drug use with negative impacts on HIV outcomes among PWID (1), in the 83 countries with data, at least 67 (81%) consider drug use or consumption or possession of drugs for personal use a criminal offense, and nine consider it a noncriminal offense (16). A global estimate found that 58.4% of PWID had a lifetime history of incarceration (3) The fear of arrest and prosecution dissuades people from seeking and using services (1), and access to services during periods of arrest may be limited or nonexistent.

Another commonly cited barrier to care is economic distress*,* including homelessness or transient housing (8, 13). Globally, almost a quarter of PWID have experienced homelessness or unstable housing within the past year (17).

In addition, the lack of financial means for communication and transportation (8, 13) contributes to the layers of barriers faced by PWID, underscoring the need to offer a comprehensive package of services at service delivery points to address multifaceted economic barriers and enable access to all services in one trip.

HIV programs have seen some success in addressing these barriers and reaching previously unreached PWID with services through empowering community-led organizations to implement peer-led, community-based, decentralized, and differentiated service delivery models combining psychosocial and paralegal support and enabling environment strategies, such as engaging and sensitizing law enforcement officers to prevent arrests and harassment of clients accessing services (18–21). Addressing structural barriers to HIV care services and empowering PWID community leadership is necessary to close the equity gaps in health outcomes.

## Gender disparities and other intersectionality of vulnerabilities

Women who inject drugs are disproportionately affected by both HIV and HCV (22). While men are five times more likely than women to inject drugs, women who inject drugs are 1.2 times more likely to be living with HIV than men who inject drugs. Women who inject drugs face additional barriers, such as gender inequalities, increased likelihood of gender-based violence, and fear of having their children removed from their care, increasing their HIV prevalence rate compared to men who inject drugs (1, 2, 8).

Some women who inject drugs also sell or exchange sex. Globally, an estimated 14.9% of women who inject drugs have recently engaged in sex work (3). They are also likely to have intimate partners who also inject drugs; to experience physical assault or rape; and, compared to men, to experience abuse from law enforcement officers and intimate partners that occurs with impunity (2). Due to their increased likelihood of contact with law enforcement and, in many contexts, the criminalization of sex work, their possibility of arrest increases. These experiences compound their barriers to accessing care and the likelihood of acquiring HIV both sexually and through sharing needles and syringes.

Although based on limited data, a recent global estimation found that 0.2%–30.6% (average of 9%) of PWID identify as lesbian, gay, or bisexual, and 0.4% as transgender (range of 0%–2.5%) (3). As such, intersectionality between PWID and other key and priority populations amplifies their barriers to PrEP access.

## Increasing access to choice for PWID in the context of new PrEP product introduction

New PrEP products, including long-acting injectable cabotegravir (CAB PrEP) and the dapivirine vaginal ring (PrEP ring), introduce new opportunities for people with a higher likelihood of acquiring HIV. However, to improve the overall health of PWID, equal and sufficient access to these services must be assured. Women who inject drugs should also be specifically included in trials that involve the development of the dual prevention pills and other new HIV prevention technologies.

## Current gaps in research for new PrEP products’ efficacy and in studies for PWID

Research on PrEP products has generally excluded injection drug use behavior, and the trend persists in recent studies. At the time of development of this article, among the 147 studies for PrEP products in the pipeline, four included PWID specifically as a target population, one of which was a landscaping study for CAB PrEP, two CAB PrEP implementation studies in Vietnam and the United States, and one clinical phase 2 trial for lenacapavir in the United States (23). While the focus on PWID in these specific studies is promising, given the significant contributions of new HIV acquisitions among this population annually, more studies are needed to generate evidence and expand their access to HIV prevention options. This includes conducting implementation science studies with PWID and ensuring their meaningful engagement in the planning and implementation of demonstration projects or pilots for rollout across countries. Therefore, ongoing efforts to introduce new prevention products—especially CAB PrEP and PrEP ring—through demonstration projects and direct implementation in some countries must take into consideration the inclusion of PWID and other key and priority populations.

While the PrEP ring does not protect against acquiring HIV through anal sex or parenterally, women who inject drugs, including those who sell sex, also have a likelihood of acquiring HIV through vaginal sex. Yet, there has been no research to date on implementing the PrEP ring with these population groups. The engagement of women who inject drugs, including those who sell sex, in further research is needed to understand and consider their values and preferences for acceptable and preferred modalities of delivery. Further, using the PrEP ring in combination with other prevention interventions, as well as intermittent use of the PrEP ring, warrants further studies, which could also include moving from oral PrEP to the PrEP ring and back again according to circumstances (24).

The World Health Organization acknowledges it is unclear whether CAB PrEP is efficacious at preventing parenteral HIV acquisition but clarifies that PWID will benefit from CAB PrEP for sexual exposure (25). At the same time, the efficacy of CAB PrEP and its use is not yet demonstrated among PWID, and drug interactions with commonly used drugs and medications for OAT are unknown (26). Studies that have demonstrated the efficacy of these new PrEP products excluded people who had injected drugs within 90 days before enrollment and did not provide outcome measures to guide their use among PWID. PWID cannot be excluded from HIV research, and specific considerations for PWID in research methods should be addressed.

Although prioritization of PWID for new products in research lags, some studies have shown that PWID find injectable and long-acting options acceptable (27, 28), and other studies have found a preference for long-acting options (29–31), including specifically among women who inject drugs (32, 33). However, most of these studies were conducted in North America, and research gaps remain for perspectives among PWID in other contexts as well as in comparing perspectives among PWID, not only between oral PrEP and long-acting options, but also with NSP. Moreover, with the indications of acceptability and preference among PWID for long-acting options, further delays in useful evidence generation to inform scale-up must be prevented while prioritizing fostering trust with the PWID community. This requires the meaningful engagement of the PWID community in research—from conceptualization through the implementation and interpretation of results—to ensure that the evidence generated is valuable for the PWID community and can be practically applied towards safe, person-centered, acceptable, accessible, and equitable scale-up.

## Decentralizing, de-medicalizing, and integrating long-acting PrEP for PWID

As with oral PrEP, acceptable new prevention product delivery modalities are also necessary to optimize accessibility and reach for this key population. PrEP ring, CAB PrEP, and other long-acting options could be welcome options for PWID, as they could help overcome the challenges of adherence and persistence and benefit those who may not have secure locations to store drug commodities (14, 28). However, PWID have also cited barriers or concerns. Specifically, regarding CAB PrEP, worries included keeping appointments every two months as well as mistrust of health care providers, which may also apply to other long-acting options. Suggestions to address these challenges included frequent reminders, provision of longer-lasting injectable and implantable PrEP, and distribution through local pharmacies (14, 28). Addressing the mistrust of health care providers by PWID will require a multifaceted approach, including ongoing sensitization and orientation of providers around the specific needs of PWID, as well as ensuring PWID are adequately engaged in the design, implementation, and monitoring of programs and research studies. Centralized and medicalized delivery of long-acting HIV prevention options will possibly be met by challenges in reaching PWID.

Long-acting HIV prevention options for PWID should be integrated with the provision of other HIV prevention and HR services through community-based service delivery modalities to improve access. The provision of the options should also be implemented with meaningful engagement of the PWID community. The PrEP ring, which can be self-inserted with counseling from a trained provider, can feasibly be provided through community-based modalities. Yet, other options may be more challenging. For example, CAB PrEP is an intermuscular gluteal injection, so its provision may be restricted due to the need for qualified people and appropriate settings to administer it. Strategies for decentralizing the delivery of new prevention products must be explored in consultation with PWID—including specifically women who inject drugs and those who also sell sex—to ensure equitable access for all populations. It is promising to see studies exploring differentiated administration of CAB PrEP, such as alternative injection sites on the body (34), which may open possibilities of a wider range of locations for administration. Lencapavir, which has been studied as a subcutaneous injection, should also be considered. It may eventually be eligible for self-injection, potentially providing greater flexibility for decentralized distribution (35).

## Discussion

While new PrEP products, like the PrEP ring and CAB PrEP, introduce new opportunities for PWID, equity in access must be assured by addressing known barriers in service delivery and gaps in evidence to optimize impact for achieving epidemic control. Future efforts must rest on what has been learned from experience to avoid the same mistakes of insufficient scale-up of proven HR options, including NSP, OOM, OAT, and condom distribution; lack of coordination between HIV prevention and other HR options; inadequate engagement and empowerment of the PWID community to understand their values and preferences and inform service delivery models and needs; and exclusion of PWID from research.

Programs can learn from the successes and failures in reaching PWID and increasing coverage of services through community-led implementation of differentiated and comprehensive service delivery models. New HIV prevention product implementation must be clearly framed as an option within the HR service package including NSP, OOM, and OAT, and not as their replacement, and should emphasize the offering and direct provision of the comprehensive package. Implementation tools, trainings, and standard operating procedures should be adapted specifically for PWID and include NSP, OAT, OOM, condoms and other HR strategies specific to PWID as options of care. All offerings should be provided through integrated health care and broader social services to address intersecting health care needs and improve efficiency and equitable access to complementary services.

Strategies to address barriers among subpopulations must also be incorporated. Investments are needed to address the well-known yet enduring access barriers for PWID including, but not limited to, stigma, discrimination by health care providers, counterproductive law enforcement practices, criminalization, transient housing, and lack of financial means. Implementation should address these socioeconomic barriers by engaging and sensitizing local law enforcement to avoid arrests and harassment of clients, health care workers, and other program stakeholders; training health care providers on PWID-friendly services; empowering peer community health workers to support appointment keeping; and providing access to broader health and social services. Gender disparities must not be neglected and should be addressed through engaging women who inject drugs and those who also sell sex in planning for and integrating services with other women-centered interventions, including options for sexual and reproductive health and violence response services.

PWID, including specifically women who inject drugs and those who also sell sex, cannot be excluded from research on new PrEP products, and research must address the practicality of implementation given the known barriers to the generalizability of implementation recommendations. More research engaging the PWID community is needed in various contexts globally to understand their values and preferences for HIV prevention and HR options and the preferred and acceptable service delivery models for addressing their various circumstances. Exploration on service delivery modalities informed by PWID are needed to allow for tailored and simplified provision of the PrEP ring, CAB PrEP, and other long-acting options tailored for PWID alongside HR services to ensure equal access.

The PWID community has long been identified as a group with a high burden of the HIV epidemic and they continue to contribute disproportionately to global new HIV acquisitions. However HIV prevention services have not been optimally scaled among this population. The key lessons described should be well-acknowledged and be deliberately applied to future HIV prevention programming to rapidly close the equity gaps. The strong and persistent engagement and empowerment of the PWID community and organizations is critical and cannot be neglected to ensure research and programming continue to remain acceptable, equitable, and relevant as well as to ensure the sustainability of such programming.

While waiting for necessary additional evidence and approvals, programs should work together with the PWID community in research, implementation, and decision-making to expand, prioritize, and facilitate efforts and investment and increase equal access to all preferred and recommended HIV, HR, broader health, and social services. These efforts should also improve the overall health among PWID through strategies for an enabling environment and continuous building of the trust necessary for acceptable rollout of new PrEP products.
